# The complete mitochondrial genome of the chicken roundworm *Ascaridia galli* (Nematoda: Ascaridiidae)

**DOI:** 10.1080/23802359.2023.2261638

**Published:** 2023-10-03

**Authors:** Yujun Shuai, Qiqi Xue, Minghui Zou, Jinhong Zhao

**Affiliations:** Department of Medical Parasitology, Wannan Medical College, Wuhu, China

**Keywords:** *Ascaridia galli*, mitogenome, phylogenetic analysis

## Abstract

*Ascaridia galli* (Nematoda: Ascaridiidae), infecting mainly the small intestine of chickens, is one of the most common nematodes in poultry worldwide. The complete mitochondrial genome sequence of *A. galli* was 13,981 bp in total length with 36 coding genes, namely, 12 protein-coding genes (PCGs), two ribosomal RNAs, and 22 transfer RNAs. All PCGs were transcribed in one direction. Phylogenetic analysis of the mitogenome of *A. galli* would further contribute to resolving its phylogenetic position and offer novel perspectives on phylogenetic studies of *A. galli*.

## Introduction

*Ascaridia galli* (Schrank, 1788) (Nematoda: Ascaridiidae), is the largest avian nematode in the Ascaridiidae family and one of the most common nematodes in poultry worldwide, infecting mainly the small intestine of chickens (Urbanowicz et al. 2018). *A. galli* can lead to anorexia, weight loss, bleeding of the intestinal mucosa, alteration of hormone levels, and even death for the host (Permin et al. 2006), thus negatively affecting the economic development of the livestock and poultry industries. In cases of genetic isolation or interpopulation hybridization, the structure of mitochondrial DNA (mtDNA) tends to remain unchanged compared to that of autosomally inherited DNA, and therefore, mtDNA is the main source of markers for population inheritance and evolutionary studies (Shao and Barker 2007; Muchadeyi et al. 2008). This study was undertaken to better understand the biome of *A. galli* and to provide new insights.

## Materials and methods

Parasite specimens were collected from the small intestine of a domestic chicken in Shexian County (N29°51′, E118°24′), Huangshan City, Anhui Province, China. After collection, the nematodes were repeatedly rinsed with 0.9% normal saline and stored in 70% alcohol solution. They were identified as female *A. galli* according to morphological and molecular characteristics (Tian-yu et al. 2020). A specimen was deposited at the Department of Parasitology, Wannan Medical College, Anhui Province, China (Jinhong Zhao, zhaojh@wnmc.edu.cn), under voucher number WNMC-I-108.

**Figure 1.**
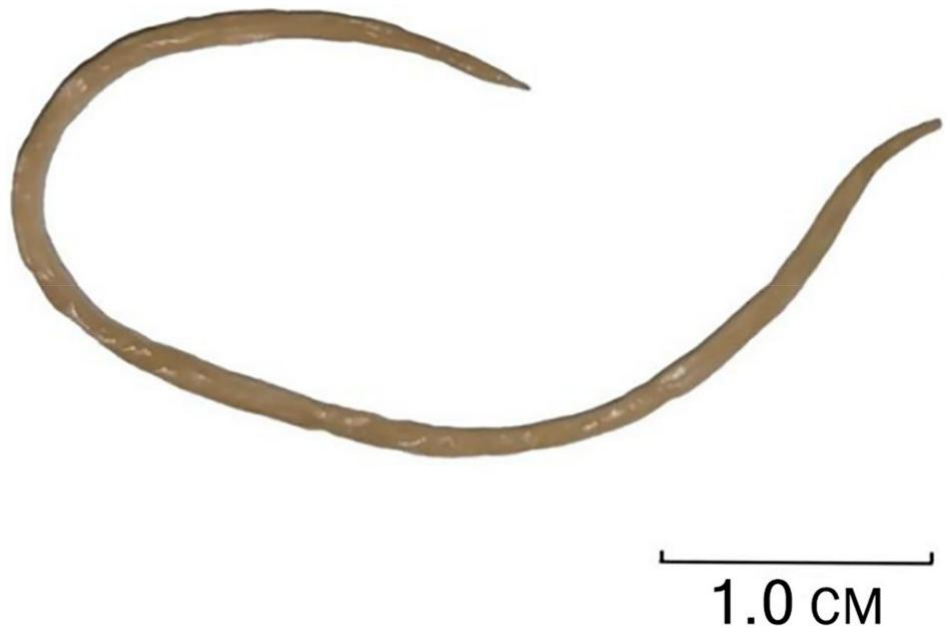
Reference image of *Ascaridia galli* collected from Shexian County, Anhui Province, China. The photo was taken by Yujun Shuai.

The *A. galli* female body is yellowish white and cylindrical, with a straight tail tip and a total length of 5.9 cm in the study (Figure 1). Total genomic DNA was extracted from a single *A. galli* sample using the TIANamp Genomic DNA Kit (TIANGEN, Beijing, China) according to the manufacturer’s instructions. For total genomic DNA sequencing, Sanger sequencing was performed by GENERAL BIOL Co. (Chuzhou, China). The complete genome sequence was archived in the GenBank DNA database under accession no. OQ286042. The mitochondrial genome map of *A. galli* was constructed using the CGView Server (Grant and Stothard 2008) (http://cgview.ca).

To further investigate the phylogenetic relationships of *A. gall*, a maximum-likelihood (ML) tree was established by 12 protein-coding genes (PCGs) of 24 nematode mitochondrial genomes downloaded from GenBank. Phylogenetic analyses were conducted using ML methods with *Wuchereria bancrofti* (GenBank accession no. NC016186) as the outgroup.

## Results

**Figure 2.**
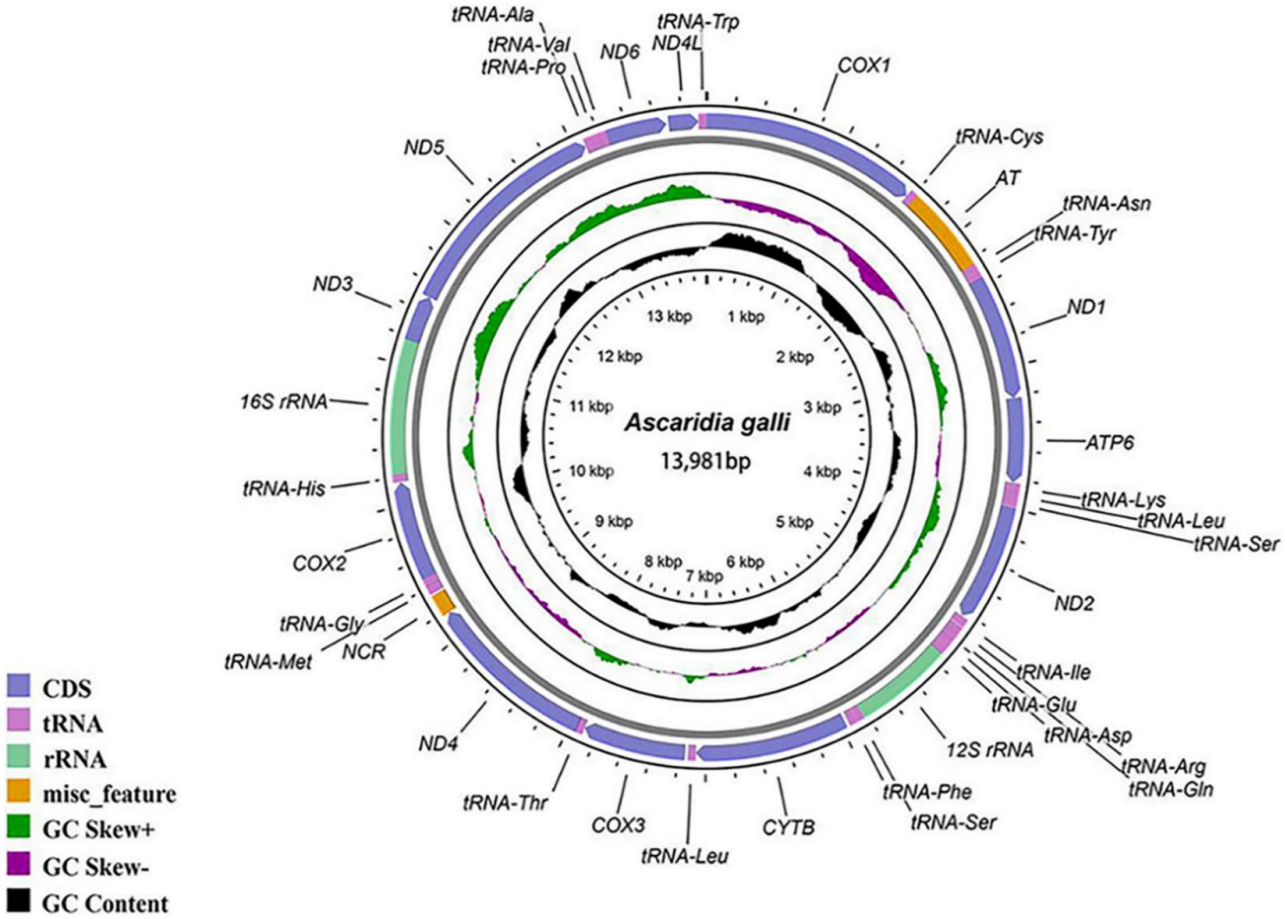
Mitochondrial genome map of *Ascaridia galli* constructed in this study.

The mtDNA size of *A. galli* (GenBank accession no. OQ286042) was 13,981 bp, with an A + T bias of 76.10% and 12 PCGs, 22 transfer RNA genes (tRNAs), two ribosomal RNA genes (rRNAs), and two noncoding regions (NCRs) (Figure 2). Similar to the findings in most other nematodes, the atp8 gene is not present in the mt genome of *A. galli*, and the transcription of all mitochondrial genes is unidirectional (Liu et al. 2013). Twelve PCGs, except *cytb*, *cox*3, and *nad*4L, were inferred to use the incomplete stop codon ‘T’, and the remaining nine genes (*cox*1, *nad*1, *atp*6, *nad*2, *nad*4, *cox*2, *nad*3, *nad*5, and *nad*6) were predicted to use the typical stop codon ‘TAG’ or ‘TAA’. Twenty-two tRNAs ranged in length from 51 bp (tRNA-ser) to 63 bp (tRNA-Met). For the two rRNAs, the small subunit rRNA (*rrn*S) between tRNA-Glu and tRNA-Ser had a length of 700 bp, and the large subunit rRNA (*rrn*L) located between tRNA-His and *nad*3 had a length of 955 bp. Regarding the two NCRs, the NC1 region (also regarded as the AT-rich region, 615 bp) and NC2 (158 bp) were located between tRNA-Cys and tRNA-Asn and between nad4 and tRNA-Met, respectively.

**Figure 3.**
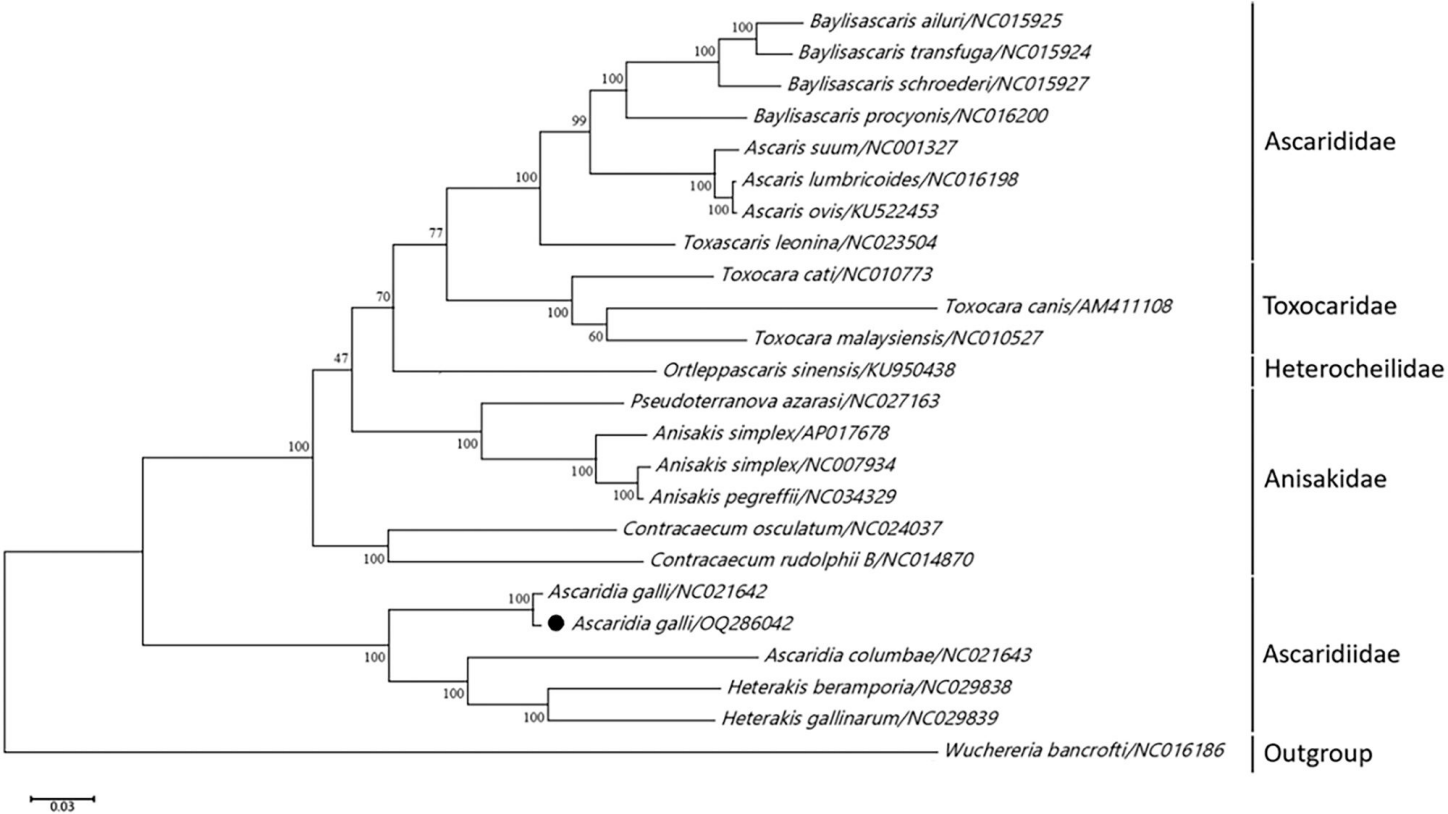
Maximum-likelihood (ML) tree built with concatenated nucleotide sequences of 12 mitochondrial PCGs of *A. galli* and other relevant nematode parasites, utilizing MEGA X software with 10,000 bootstrap replications. The filled black circle indicates the nematode studied here.

The ML phylogenetic tree showed that all the nematodes of the order Ascaridida belonged to five families: Anisakidae, Ascarididae, Toxocaridae, Heterocheilidae, and Ascaridiidae. *A. galli* was highly homologous to *Ascaridia galli* (GenBank accession no. NC021642) in GenBank, forming a monophyletic group belonging to the family Ascaridiidae. Moreover, we found that the results of phylogenetic analyses obtained using Bayesian inference models were similar. The chicken roundworm examined in this study had high bootstrap values for inclusion in Ascaridiidae (Figure 3).

## Discussion and conclusions

In the present study, the complete mitogenome of *A. galli* was assembled and analyzed. The mitochondrial genome of *A. galli* is 13,981 bp in length and expresses high AT bias. Phylogenetic analysis was also performed. We expect that these data will be conducive to providing new references and insight for studies on the phylogenetics and genetics of *A. galli*.

## Supplementary Material

Supplemental Material

Supplemental Material

Supplemental Material

## Data Availability

The genome sequence data that support the findings of this study are openly available in GenBank of NCBI at https://www.ncbi.nlm.nih.gov/nuccore/OQ286042.
