# Supplementary material for: The complete mitochondrial genome of the chicken roundworm Ascaridia galli (Nematoda: Ascaridiidae)
Source: Mitochondrial DNA B Resour. 2023 Oct 3;8(10):1029–31. doi: 10.1080/23802359.2023.2261638 (PMC10552605; doi:10.1080/23802359.2023.2261638)
Supplement: Supplemental Material [file TMDN_A_2261638_SM1229.docx]

| Gene | Position | Size | Spacer(+)  Overlap(-) | Start codon | Stop codon | AA |
| --- | --- | --- | --- | --- | --- | --- |
| *cox1* | 1-1563 | 1563 | 1(+) | ATG | TAA | 519 |
| *tRNA-Cys* | 1565-1620 | 56 | 0 |  |  |  |
| *AT* | 1621-2235 | 615 | 0 |  |  |  |
| *tRNA-Asn* | 2236-2389 | 54 | 1(+) |  |  |  |
| tRNA-Tyr | 2291-2349 | 59 | 3(-) |  |  |  |
| *Nad1* | 2350-3222 | 873 | 1(-) | TTG | TAA | 289 |
| *ATP6* | 3223-3818 | 597 | 2(+) | ATA | TAA | 197 |
| *tRNA-Lys* | 3821-3881 | 61 | 1(-) |  |  |  |
| tRNA-Leu | 3881-3936 | 56 | 0 |  |  |  |
| tRNA-Ser | 3937-3987 | 51 | 3(+) |  |  |  |
| nad2 | 3991-4833 | 843 | 3(+) | TTG | TAG | 279 |
| *Trna-Ile* | 4837-4896 | 60 | 4(+) |  |  |  |
| tRNA-Arg | 4901-4956 | 56 | 0 |  |  |  |
| tRNA-Gln | 4957-5011 | 55 | 1(-) |  |  |  |
| tRNA-Asp | 5011-5070 | 60 | 1(+) |  |  |  |
| tRNA-Glu | 5072-5132 | 61 | 3(-) |  |  |  |
| *rrnS* | 5130-5829 | 700 | 2(+) |  |  |  |
| tRNA-Ser | 5832-5886 | 55 | 2(+) |  |  |  |
| *tRNA-Phe* | 5889-5945 | 57 | 24(+) |  |  |  |
| cytb | 5970-7071 | 1102 | 0 | ATG | T | 366 |
| tRNA-Leu | 7074-7127 | 54 | 21(+) |  |  |  |
| cox3 | 7149-7893 | 745 | 0 | TTG | T | 247 |
| tRNA-Thr | 7894-7949 | 56 | 6(-) |  |  |  |
| nad4 | 7944-9179 | 1236 | 0 | GTG | TAG | 410 |
| NCR | 9180-9337 | 158 | 13(+) |  |  |  |
| tRNA-Met | 9351-9413 | 63 | 1(-) |  |  |  |
| tRNA-Gly | 9413-9468 | 56 | 0 |  |  |  |
| cox2 | 9469-10164 | 696 | 4(+) | TTG | TAG | 230 |
| tRNA-His | 10169-10224 | 56 | 8(-) |  |  |  |
| *rrnL* | 10217-11172 | 955 | 1(-) |  |  |  |
| nad3 | 11172-11507 | 336 | 1(-) | TTG | TAA | 110 |
| nad5 | 11507-13087 | 1581 | 1(+) | ATA | TAG | 525 |
| *tRNA-Ala* | 13089-13143 | 55 | 0 |  |  |  |
| tRNA-Pro | 13144-13201 | 58 | 1(-) |  |  |  |
| tRNA-Val | 13201-13256 | 56 | 0 |  |  |  |
| nad6 | 13257-13691 | 435 | 14(+) | TTT | TAA | 143 |
| Na4L | 13706-13922 | 217 | 0 | ATT | T | 71 |
| *tRNA-Trp* | 13923-13980 | 58 | 1(+) |  |  |  |

AT: AT rich region NCR: Non-coding region.
