# Supplementary material for: The complete mitochondrial genome of the chicken roundworm Ascaridia galli (Nematoda: Ascaridiidae)
Source: Mitochondrial DNA B Resour. 2023 Oct 3;8(10):1029–31. doi: 10.1080/23802359.2023.2261638 (PMC10552605; doi:10.1080/23802359.2023.2261638)
Supplement: Supplemental Material [file TMDN_A_2261638_SM1228.docx]

| Table 1 Primers for mitochondrial genome of *Ascaridia galli* | | |  |
| --- | --- | --- | --- |
|  |  |  |  |
| Primers | F/R | Primer sequence (5’-3’) |  |
|  |  |  |  |
| YW1 | F | GGTGGTCTCACTGGTGTTAT |  |
|  | R | CAGAACAAACCACTCCAAAT |  |
|  |  |  |  |
| YW2 | F | GTTGTTGAAGAAGGAGCAGG |  |
|  | R | AATGCAAGGGAGAAACAC |  |
|  |  |  |  |
| YW3 | F | ATTGGTGTTTCTCCCTTG |  |
|  | R | GGATGATTTGTACCGTTCT |  |
|  |  |  |  |
| YW4 | F | GAGAACGGTACAAATCATCC |  |
|  | R | CGGGAACAATGTGAATAGG |  |
|  |  |  |  |
| YW5 | F | TATTATGGCTGGTACTACTTTG |  |
|  | R | CTGAAATCATAACAACACCC |  |
|  |  |  |  |
| YW6 | F | TGGGTGTTGTTATGATTTCAG |  |
|  | R | CTACCAAAGGAATACTAAAGCC |  |
|  |  |  |  |
| YW7 | F | GACTAATGCTGTTATGGGAAAG |  |
|  | R | ATCAACAGAATTTCCGAAGAC |  |
|  |  |  |  |
| YW8 | F | GAGGGCATAAAAGTAGCAAA |  |
|  | R | GTATTCATAGCACCACTACAACT |  |
|  |  |  |  |
| YW9 | F | TGAGATAGTTGTAGTGGTGCT |  |
|  | R | AGGCACCTGTATCAAGTTAG |  |
|  |  |  |  |
| YW10 | F | TTGTTGCTGGTGTTTATGTC |  |
|  | R | GGAGCACCTAACAGCAAAG |  |
|  |  |  |  |
| YW11 | F | TTGTCTGATGGGCAGTTGT |  |
|  | R | AAACAGCACCCATACTCAAC |  |

**M**
