# Supplementary material for: The complete mitochondrial genome of the chicken roundworm Ascaridia galli (Nematoda: Ascaridiidae)
Source: Mitochondrial DNA B Resour. 2023 Oct 3;8(10):1029–31. doi: 10.1080/23802359.2023.2261638 (PMC10552605; doi:10.1080/23802359.2023.2261638)
Supplement: Supplemental Material [file TMDN_A_2261638_SM1227.docx]

Figure1 PCR gel image of 11 pairs of primers in *Ascaridia galli* mitochondrial genome.


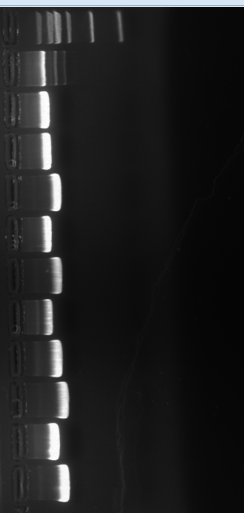


**1197654**

**1097654**

**97654**

**87654**

**77654**

**6654**

**554**

**44**

**33**

**2**

**1**

**M**

M: DNA Maker, contains a mix of 6 individual DNA fragments (in base pairs): 100, 250, 500, 750, 1000, 2000 bp.

Number 1-10 means target genes, from left to right was primer YW1 to primer YW11, respectively.

No: Primers: Amplified mitochondrial gene fragments

1 YW1: cox1-nad1

2 YW2: nad1-nad2

3 YW3: nad2-rrnS

4 YW4: rrnS-cytb

5 YW5: cytb-nad4

6 YW6: nad4-nad4

7 YW7: nad4-rrnL

8 YW8: rrnL-nad5

9 YW9: nad5-nad5

10 YW10: nad5-cox1

11 YW11: cox1-cox1
